# Construction of homozygous diploid potato through maternal haploid induction

**DOI:** 10.1007/s42994-022-00080-7

**Published:** 2022-09-15

**Authors:** Jinzhe Zhang, Jian Yin, Jiayi Luo, Die Tang, Xijian Zhu, Jie Wang, Zhihong Liu, Pei Wang, Yu Zhong, Chenxu Liu, Canhui Li, Shaojiang Chen, Sanwen Huang

**Affiliations:** 1 Institute of Vegetables and Flowers, Chinese Academy of Agricultural Sciences, Key Laboratory of Biology and Genetic Improvement of Horticultural Crops of the Ministry of Agriculture, Sino-Dutch Joint Laboratory of Horticultural Genomics, Beijing, 100081 China; 2 Shenzhen Branch, Guangdong Laboratory of Lingnan Modern Agriculture, Genome Analysis Laboratory of the Ministry of Agriculture and Rural Affairs, Agricultural Genomics Institute at Shenzhen, Chinese Academy of Agricultural Sciences, Shenzhen, 518120 China; 3 The AGISCAAS-YNNU Joint Academy of Potato Sciences, Yunnan Normal University, Kunming, 650500 China; 4 National Maize Improvement Center of China, Key Laboratory of Crop Heterosis and Utilization/Engineering Research Center for Maize Breeding, Ministry of Education, College of Agronomy and Biotechnology, China Agricultural University, Beijing, 100193 China

**Keywords:** Doubled haploid technology, Haploid induction, Diploid potato, Inbred lines, *StDMP*

## Abstract

Reinventing the tetraploid potato into a seed-propagated, diploid, hybrid potato would significantly accelerate potato breeding. In this regard, the development of highly homozygous inbred lines is a prerequisite for breeding hybrid potatoes, but self-incompatibility and inbreeding depression present challenges for developing pure inbred lines. To resolve this impediment, we developed a doubled haploid (DH) technology, based on mutagenesis of the potato *DOMAIN OF UNKNOWN FUNCTION 679 membrane protein* (*StDMP*) gene. Here, we show that a deficiency in *StDMP* allows the generation of maternal haploids for generating diploid potato lines. An exercisable protocol, involving hybridization, fluorescent marker screening, molecular and flow cytometric identification, and doubling with colchicine generates nearly 100% homozygous diploid potato lines. This *dmp*-triggered haploid induction (HI) system greatly shortens the breeding process and offers a robust method for generating diploid potato inbred lines with high purity.

**Supplementary Information:**

The online version contains supplementary material available at 10.1007/s42994-022-00080-7.

Dear Editor,

Potato (*Solanum tuberosum* L.) is the world’s most important tuber crop (Stokstad 2019). Unlike many other major crops, the complexity of tetrasomic inheritance, and reliance on clonal propagation, greatly hamper genetic improvement of cultivated potatoes. An increasing number of studies have focused on reinventing the potato as an inbred-line-based diploid seed crop, also referred to as the hybrid potato, which would transform potato breeding from being slow and non-accumulative to being fast and iterative (Lindhout et al. 2011; Jansky et al. 2016; Li et al. 2022; Zhang et al. 2021).

To generate a hybrid with sufficient heterosis and uniformity, the existence of pure inbred lines with high genome homozygosity is crucial. However, self-incompatibility and inbreeding depression represent impediments for the development of pure inbred potato lines (Ye et al. 2018; Zhang et al. 2019; Zhou et al. 2020). Initial efforts, through genome design and five generations of self-pollination, yielded inbred lines with high homozygosity (Zhang et al. 2021). Notably, Solyntus, a self-compatible diploid inbred line, developed by researchers at the Solynta Company and Wageningen University, still showed heterozygosity over > 20% of the genome, even after over a decade of self-pollination and selection (van Lieshout et al. 2020). Clearly, potato hybrid breeding is still at an early stage, and there remains an urgent need to develop an efficient method for obtaining pure inbred lines.

Compared with conventional plant breeding approaches, which require many generations of self-pollination and/or backcrossing, doubled haploid (DH) production enables the fixation of haploid genomes within inbred lines in a single generation (Jacquier et al. 2020). Haploid gametophytes can be induced to develop, as DH sporophytes, through in vitro tissue culture, in vivo interspecific hybridization and intraspecific hybridization with inducer lines, or manipulation of CENH3 centromeric histone proteins (Coe 1959; Seguí-Simarro and Nuez 2007; Ravi and Chan 2010). Among these methods, in vivo triggered haploid induction (HI) is the most efficient method. Indeed, several elite tetraploid cultivars were already diploidized by several accessions of Andigenum group potatoes (Hutten et al. 1993). However, as ~ 70% of the natural potato germplasm is diploid (Spooner et al. 2014), their induction to yield the haploid state would represent a highly efficient way to develop pure inbred lines.

Recently, significant breakthroughs in DH production were achieved, based on the identification of two *Zea mays* HI genes, *Phospholipase-a1*/*Matrilineal/Not like dad* (*ZmPLA1*/*MTL*/*NLD*) and *Domain of unknown function 679 membrane protein* (*ZmDMP*) (Kelliher et al. 2017; Liu et al. 2017; Yao et al. 2018; Zhong et al. 2019, 2020, 2021; Wang et al. 2021). *ZmPLA1*/*MTL*/*NLD* homologs have been identified only in monocots (Kelliher et al. 2017; Yao et al. 2018), but *ZmDMP* homologs were identified in both monocots and dicots (Zhong et al. 2020). Although the utility of *dmp* mutants, for HI production, has been demonstrated in some dicot crops (Zhong et al. 2020, 2021, 2022; Wang et al. 2021), it remains unknown whether this approach is applicable for potato.

A single *DMP* ortholog, *StDMP* (Soltu.DM.05G005100), is present in the potato genome (Zhong et al. 2020). A protein comparison between StDMP, ZmDMP, AtDMP8, AtDMP9, and SlDMP revealed that the entire DUF679 domain is conserved among all of these DMP proteins, with the first predicted transmembrane domain being especially conserved (Fig. S1A). Moreover, *StDMP* is expressed exclusively in potato pollen, consistent with the *ZmDMP*, *AtDMP8, AtDMP9,* and *SlDMP* expression patterns (Fig. S1B).

The close agreement of these features suggests a deficiency in *StDMP* might induce maternal haploids in potato. To induce mutations in *StDMP*, we designed a CRISPR/Cas9 construct, targeting the first exon of *StDMP*, which also included a FAST-Red marker for haploid identification (Zhong et al. 2020) (Fig. S2A). In total, some 276 transgenic positive plantlets were generated, among which 20 individuals contained mutations in *StDMP.* However, some mutants were chimeric and others doubled their ploidy during tissue culture, which is a common phenomenon in potato (Karp et al. 1989). Fortunately, ultimately one diploid mutant clone was obtained that contained biallelic mutations in *StDMP* (Fig. S2B).

Hybridization of wild-type plants with *atdmp8 atdmp9* mutants induces maternal haploids in *Arabidopsis* (Zhong et al. 2020). To investigate whether *StDMP* dysfunction could also induce maternal haploids, upon outcrossing, two wild-type female plants, from different genetic backgrounds, were crossed with the *stdmp* mutant, including PG6359 and an F_1_ hybrid from crossing A6-26 and E4-63 (Zhang et al. 2021). Due to FAST-Red reporter (*OLEO1:OLEO1-RFP*) expression, nearly half of the hybrid seeds were red under white light (Fig. 1A), indicating the FAST-Red marker is heterozygous in the *stdmp* mutant. Under fluorescent light, the red seeds showed a strong RFP signal throughout the seed, whereas the white seeds were further divided into two groups: many white seeds showed no RFP signal, whereas some had a weak RFP signal (Fig. 1A).

**Fig. 1:**
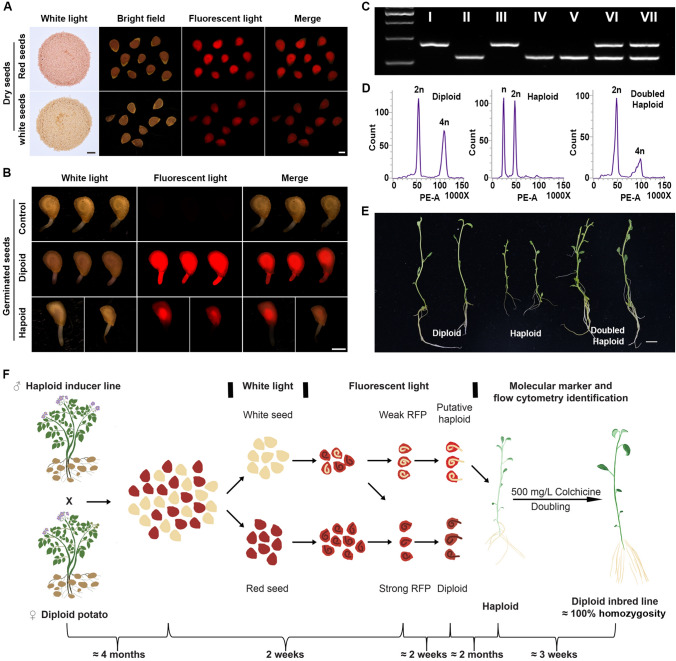
Construction of homozygous diploid potato via* stdmp*-based maternal haploid induction. **A**, **B** FAST-Red-based haploid seed identification. Under white light, the hybrid seeds could be divided into white seeds and red seeds, due to the presence of the FAST-Red marker. Some of the white seeds showed weak RFP signal under fluorescent light (**A**). During seed germination, putative haploids did not show RFP signal in the embryo root tip (**B**). **C** Seedlings from putative haploids were genotyped with a polymorphic marker between the inducer line and testers. The Roman numerals I to VII represent the bands from S15-65 (I), PG6359 (II), *stdmp* mutants (III), haploids from PG6359 × *stdmp* (IV and V), and diploids from PG6359 × *stdmp* (VI and VII). **D** Flow cytometry ploidy verification of the diploid control, putative haploid, and doubled haploid. **E** Phenotypes of the haploid, diploid, and doubled haploid. Haploid plants had smaller vegetative organs than diploid or doubled haploid plants. **F** Schematic overview of homozygous diploid potato generation, using the *stdmp* inducer line. Hybrid seeds could be easily divided into white and red seeds, under white light. Under fluorescent light, a few white seeds with weak RFP signal were selected for further analysis. Germinated seeds were checked for the absence/presence of RFP signal in the root tip. Seeds with no RFP signal in the emerging root tip are putative haploids and were further confirmed by molecular markers and ploidy analysis. True haploid plants were treated with colchicine to obtain doubled haploids. Scale bars: 1 cm (**A**, white light and **E**) and 1 mm (**A**, fluorescent light and **B**)

All seeds with a weak RFP signal were sown, but most failed to germinate (Table S1). Among the germinated seeds, the red seeds showed weak RFP signal in the endosperm and a strong RFP signal in the embryo and root tip, whereas some white seeds with weak RFP signal showed weak RFP signal in the endosperm, but lacked RFP signal in the embryo and root tip (Fig. 1B), suggesting these were maternal haploid embryos.

To test this hypothesis, we examined the genotype, phenotype, and ploidy of the seedlings derived from seeds with weak RFP signal in the endosperm. First, we developed a polymorphic marker and all putative haploids were identified with only the female genotype (Fig. 1C), consistent with their maternal haploid identities. Next, the ploidy levels of these putative haploids were confirmed by flow cytometry (Fig. 1D). Compared with diploid controls, haploid plants appeared smaller and had measurably shorter roots, smaller leaves, and narrower internodes (Fig. 1E), similar to previously described haploids (Zhong et al. 2020).

To further test the maternal origin of these haploids, four of the seedlings were used for whole-genome resequencing. Single nucleotide polymorphism (SNP) analysis showed that none of these seedlings carried paternally derived SNPs; only maternally derived SNPs from different haplotypes were detected (Fig. S3A, B), suggesting that *stdmp* can induce clean maternal haploids. Moreover, compared with the highly homozygous inbred lines developed through genome-assisted selection (Zhang et al. 2021), our haploids showed higher homozygosity, with nearly all genomic regions being homozygous (Fig. S4, S5).

Haploid plants are sterile and must undergo chromosome doubling to develop into fertile plants; this process can be chemically induced using colchicine. The haploid potato plants were propagated, clonally, in tissue culture containing 500 mg/mL colchicine, until three to four new internodes developed from the treated meristem. Finally, 3 of the 12 haploid plants underwent success conversion into DH plants, which displayed diploid cells and vigorous plant growth (Fig. 1D, E).

In total, we identified seven haploids from two crossings (Table S1). The haploid induction rate (HIR) is, therefore, relatively low in potato, approx. 0.005–0.01% (2 × True haploids/Total seeds), which may be caused by a high level of deleterious mutations in the potato genome (Zhang et al. 2019). The homozygosity of large-effect deleterious mutations and the accumulation of minor-effect deleterious mutations could lead to embryo mortality in the haploids, which may be reflected by the excessively low germination rate of the seeds having weak RFP signal (Table S1). In addition, because the FAST-Red marker is heterozygous, in the haploid inducer, only half of the haploids were successfully identified in our screen. Developing a homozygous marker would allow a more efficient identification of haploids.

This is the first report of using maternal haploid induction technology to construct homozygous diploid potato lines. Although the induction efficiency still needs to be improved, potatoes are very easily hybridized and each hybrid fruit contains hundreds of seeds. Combined with efficient and accurate screening of an RFP marker (Table S1), haploid plants can be screened out from tens of thousands of seeds, at low cost with little effort. Moreover, compared with the traditional method of obtaining homozygous inbred lines, through self-crossing, the haploid induction technique can yield diploid potato inbred lines with higher homozygosity, without the need to overcome the obstacle of self-incompatibility. A previous study reported that, if haploids survive, not only are they free from deleterious genes, but also they have a harmonious combination of genes (Uijtewaal et al. 1987), which can be reflected by vigorous growth of the DH plants (Fig. 1E).

In summary, we developed a method to obtain nearly 100% homozygous diploid potato lines, by employing hybridization, fluorescent marker screening, molecular and flow cytometric identification, and doubling with colchicine, which can be completed in approx. 8 months (Fig. 1F). Compared with the traditional self-inbred method, this method greatly shortens the breeding process and establishes a highly effective path for establishing diploid potato inbred lines with high purity.

## Materials and methods

### Amino acid sequence alignment

The full-length amino acid sequences of the *DMP* gene for each crop and model plant were obtained from corresponding genome databases (http://spuddb.uga.edu, potato; https://solgenomics.net, tomato; https://www.arabidopsis.org, *Arabidopsis*; https://maizegdb.org, maize). The full-length amino acid sequences of *DMP* genes were aligned with MUSCLE, embedded in SnapGene software (Insightful Science; available at snapgene.com). Domain and transmembrane helix predictions were performed using the Pfam database (http://pfam.xfam.org) and TMHMM-2.0 (https://services.healthtech.dtu.dk/service.php?TMHMM-2.0), respectively.

### Reverse transcription and quantitative PCR (RT-qPCR)

Total RNA was isolated from different plant tissues, using the Quick RNA Isolation Kit (HUAYUEYANG Biotechnology, Beijing, China). Each RNA sample was treated with RNase-free DNase I (HUAYUEYANG Biotechnology, Beijing, China). Reverse transcription (RT) was carried out with 0.5–1 μg of total RNA, using GoScript™ Reverse Transcriptase (Promega). PCRs were performed on an ABI 7900, using SYBR Premix (Roche), according to the manufacturer’s instructions. Three technical replicates were used to calculate the *C*_*T*_ value, and three biological replicates were analyzed. The potato *ACTIN* gene (*Soltu.DM.03G011750.2*) was used as an internal reference. All primers used in this study are listed in Table S2.

### Plasmid construction and transformation

The 19-nt single guide RNA sequence for *DMP* in the diploid clone, S15-65, was selected manually from the conserved domain and used for BLAST analysis against the potato reference DM v6.1; no other potential matches in the genome were identified. The target sequence was incorporated into the CRISPR–Cas9 vector, pKSE401, containing the RFP tag (Zhong et al. 2020). The diploid self-incompatible *S. tuberosum* group *Phureja* S15-65 clone was used in this study. Three-week-old plantlets were used for transformation. Agrobacterium-mediated transformation of potato internodes was conducted, as previously described (Ye et al. 2018). Positive transformants were screened based on growth on medium containing 50 mg/L kanamycin.

### Screening for *dmp* mutants

To detect mutations in *StDMP*, full-length *StDMP* sequences were amplified from all regenerated plantlets and sequenced. All sequences were aligned to the *StDMP* wild-type allele, with SnapGene software. As chromosome doubling occurred, at high frequency, during potato callus regeneration, the ploidy of the mutated plants was determined by flow cytometry (BD FACSDiva v7.0 Software).

### Seed germination

The collected hybrid seeds were treated with 2 mg/mL gibberellin for 48 h, disinfected with 8% sodium hypochlorite, for 2 min, and then spread on MS medium (Murashige & Skoog Basal Medium containing Vitamins 2.17 g, sucrose 15 g, and Gelzan™ CM 3.12 g in 1 L H_2_O).

### Identification of haploids

All hybrid seeds were divided into two groups, based on their color under white light. White seeds were then screened for FAST-Red expression, using a hand-held fluorescent protein excitation light source (LUYOR-3415RG; excitation wavelength 540 nm, emission wavelength 600). All seeds with weak RFP fluorescent signal were sown on half-strength Murashige and Skoog medium. Then, germinated seeds were checked for the absence/presence of RFP signal in the root tip, under a Leica SP8 confocal microscope. Seeds with weak RFP signal, which did not show RFP signal in the embryo root tip, were scored as putative haploids, and were further confirmed by molecular marker and ploidy analysis.

### Flow cytometry

Fresh seedling leaves (0.5 g) were chopped with a razor blade in 2 mL lysis buffer (45 mM MgCl_2_, 30 mM C_6_H_5_Na_3_O_7_·2H_2_O, 20 mM MOPS, and 0.1% (v/v) Triton X-100). The homogenate was filtered through a 400 μm nylon filter, followed by centrifugation (1000 r/min. at 4 °C), for 5 min, to collect the nuclei. After staining with propidium iodide, in darkness, for 20 min, the samples were analyzed using a flow cytometer (BD FACSVerse). Data were acquired and analyzed with the BD CellQuest Pro software. The first signal peak at ~ 50 (PE-A value) was used as a diploid control. Samples with the first signal peak at ~ 25 (PE-A value) were deemed to be haploids.

### Colchicine treatment of potato haploids

The haploid explants were treated on MS20 medium, containing 500 mg/mL colchicine (Cat#C3915, Sigma), until three to four new internodes developed from the treated meristem. Expanded leaf samples were taken from the top internode for ploidy analysis. Doubled haploids were then moved to MS30 medium for continued culture.

### SNP calling and breakpoint inference analysis

For each sample, ~ 10× short reads were generated, using a HiSeq X ten platform. Clean reads were mapped to DM v6.1, using BWA, and SNPs were then extracted using SAMtools and bcftools. Variants that met the following criteria were retained: (1) SNP depth ≥ 5 and ≤ 50; (2) sequencing quality ≥ 40; (3) mapping quality ≥ 30; and (4) SNPs located in non-repetitive regions. The index of heterozygous SNPs was 0.3 ≥ and ≤ 0.7, and the index of homozygous SNPs was ≤ 0.1 or ≥ 0.9. The breakpoints of crossover events were inferred by comparing the SNP similarity of haploids to two haplotypes of parental lines, using 10-kb non-overlapping sliding windows.

## Supplementary Information

Below is the link to the electronic supplementary material.

Supplementary file1 (DOCX 4123 KB)

## Data Availability

The datasets generated and/or analyzed during the current study are available from the corresponding author on request.
