# Supplementary material for: Construction of homozygous diploid potato through maternal haploid induction
Source: aBIOTECH. 2022 Sep 15;3(3):163–8. doi: 10.1007/s42994-022-00080-7 (PMC9590536; doi:10.1007/s42994-022-00080-7)
Supplement: Supplementary file 1 — Supplementary file1 (DOCX 4123 KB) [file 42994_2022_80_MOESM1_ESM.docx]

**Supplementary materials for**

**Construction of Homozygous Diploid Potato through Maternal Haploid Induction**

Jinzhe Zhang^1,ϯ^, Jian Yin^1,2,3,ϯ^, Jiayi Luo^1,2^, Die Tang^2^, Xijian Zhu^1,2,^, Jie Wang^1,2,^, Zhihong Liu^1,2^, Pei Wang^2^, Yu Zhong^3^, Chenxu Liu^3^, Canhui Li^4^, Shaojiang Chen^3^, Sanwen Huang^2,*^

^1^. Institute of Vegetables and Flowers, Chinese Academy of Agricultural Sciences, Key Laboratory of Biology and Genetic Improvement of Horticultural Crops of the Ministry of Agriculture, Sino-Dutch Joint Laboratory of Horticultural Genomics, Beijing 100081, China.

^2^. Shenzhen Branch, Guangdong Laboratory of Lingnan Modern Agriculture, Genome Analysis Laboratory of the Ministry of Agriculture and Rural Affairs, Agricultural Genomics Institute at Shenzhen, Chinese Academy of Agricultural Sciences, Shenzhen, Guangdong 518120, China.

^3^. National Maize Improvement Center of China, Key Laboratory of Crop Heterosis and Utilization/Engineering Research Center for Maize Breeding, Ministry of Education, College of Agronomy and Biotechnology, China Agricultural University, Beijing 100193, China.

^4^. The AGISCAAS-YNNU Joint Academy of Potato Sciences, Yunnan Normal University, Kunming, Yunnan 650500, China.

^ϯ^ These authors contributed equally to this study

^*^ Author for correspondence: [huangsanwen@caas.cn](mailto:huangsanwen@caas.cn)


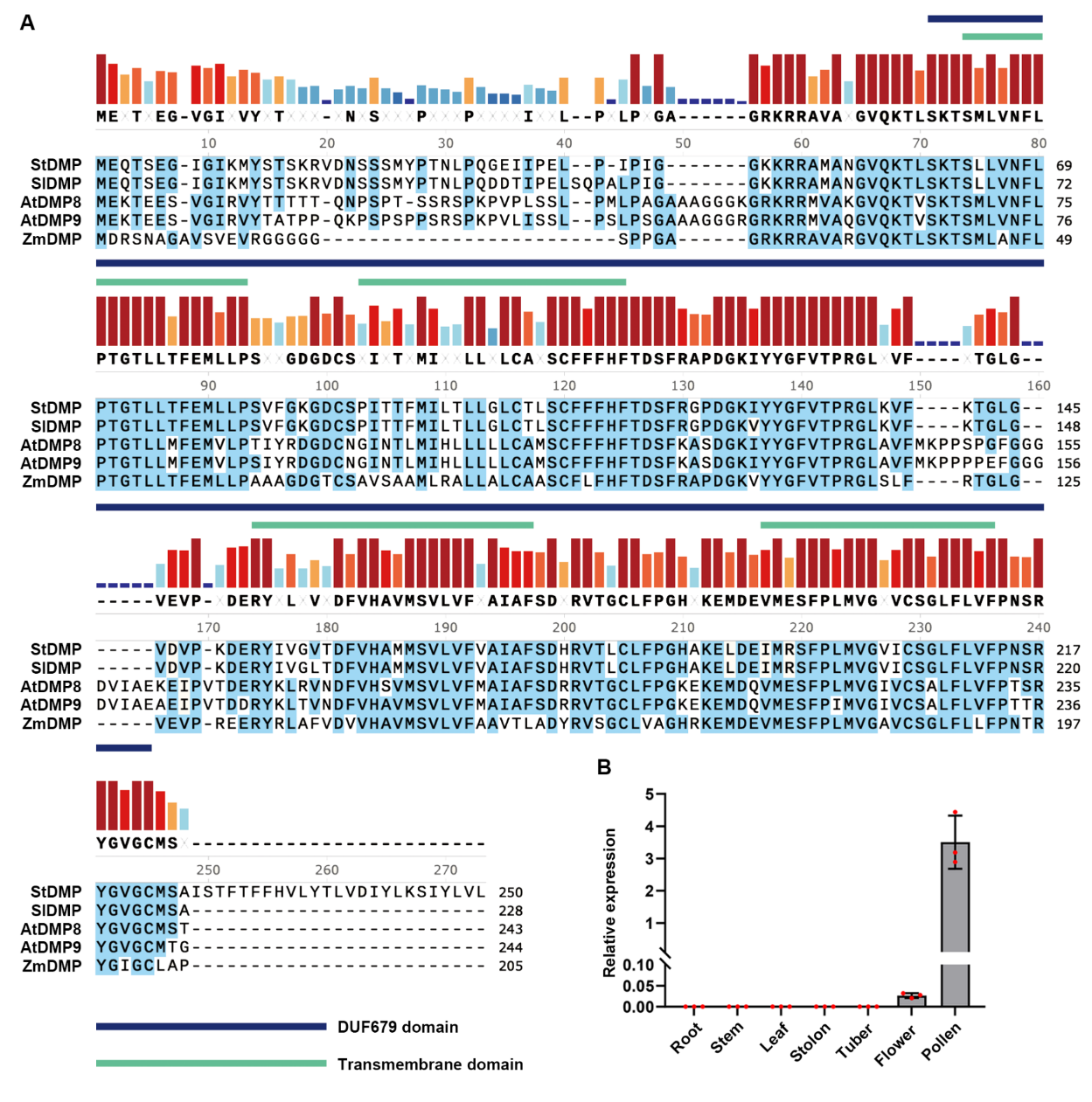


**Fig. S1.** **Amino acid alignment of *DMP* genes and the expression pattern of *StDMP***

**(A)** The alignment includes DMP proteins from potato (StDMP/Soltu.DM.05G005100.1), tomato (SlDMP/Solyc05g007920), Arabidopsis (AtDMP8/At1G09157; AtDMP9/At5G39650) and maize (ZmDMP/GRMZM2G46505). The DUF679 region is indicated by a dark blue line, and the four transmembrane domains are indicated by green lines. **(B)** Determination of relative expression levels of *StDMP* in different tissues by RT-qPCR. The relative expression was calculated using the fold change = 2^-ΔCt^ method.


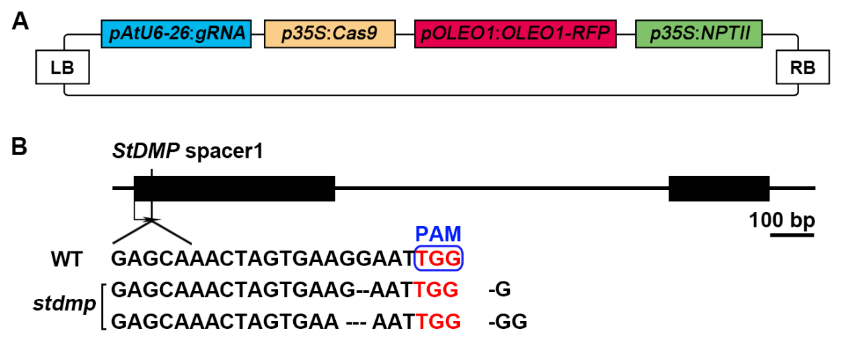


**Fig. S2. Generation of mutations in *StDMP* by CRISPR/Cas9**

**(A)** Structure of the CRISPR/Cas9 mutagenesis vector containing a single guide RNA sequence targeting *StDMP* and the FAST-Red cassette. LB, left border; RB, right border. **(B)** Sequences of wild-type *StDMP* and mutated *stdmp* are shown. The protospacer adjacent motif (PAM) sequences are indicated in red and circled in blue. Deletions are indicated by dashed lines.


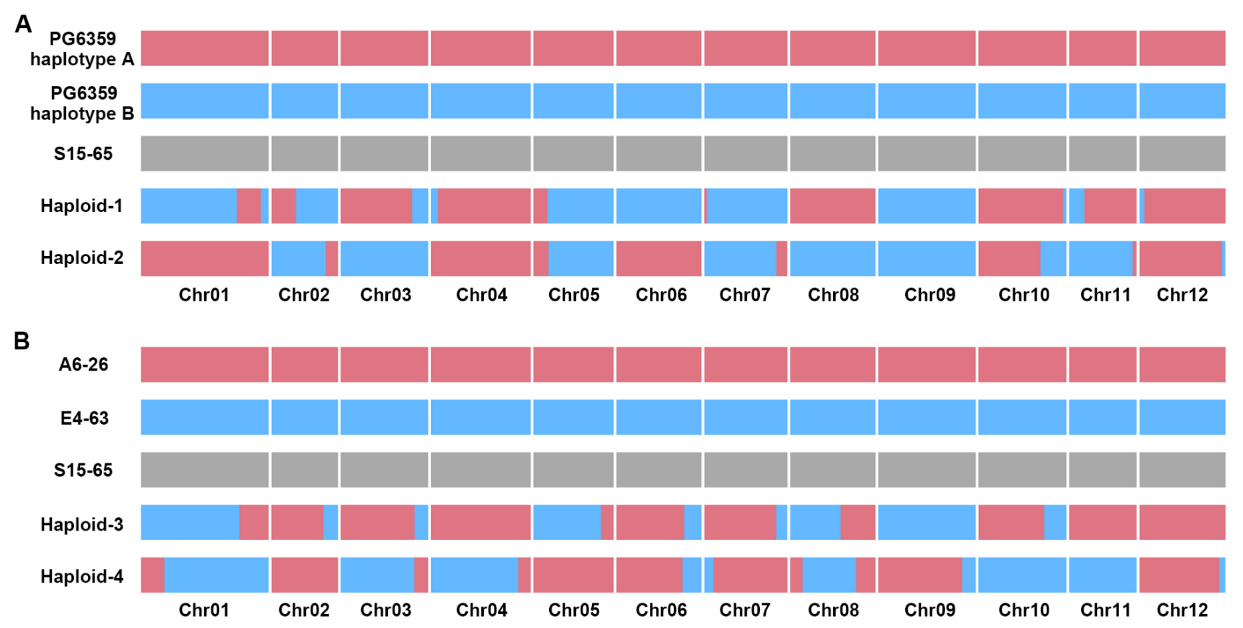


**Fig. S3.** **Recombination maps of 4 haploids and their corresponding parents**

**(A)** Haploid-1 and -2 were derived from PG6359 × *stdmp* mutant. **(B)** Haploid-3 and -4 were derived from an F1 hybrid, derived from crossing A6-26 and E4-63 × *stdmp* mutant. All recombination maps were constructed using a sliding window approach across all 12 potato chromosomes. Sample line names are indicated on the left of each recombination map.


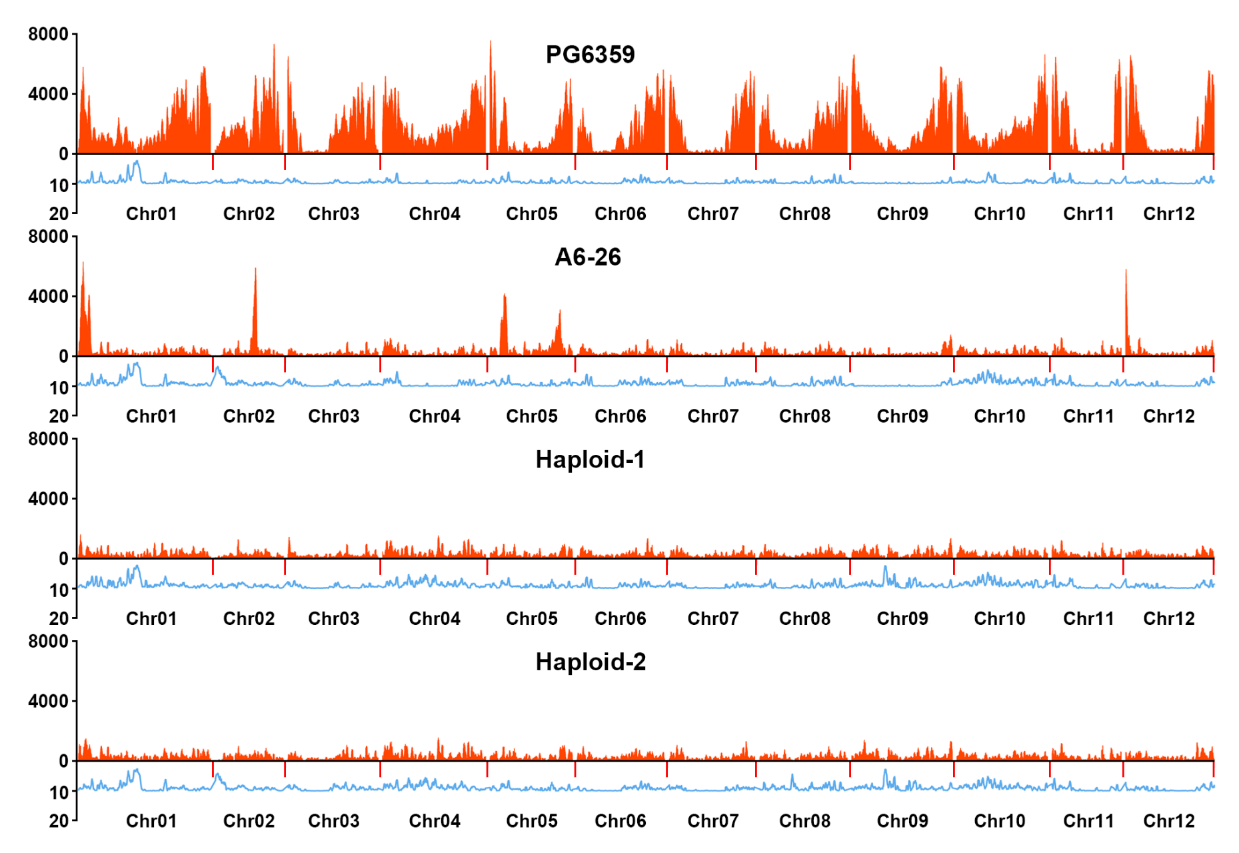


**Fig. S4. Distribution of heterozygous SNPs in haploid-1 and -2 and their corresponding maternal parent**

Chromosomal heterozygous SNP distribution maps were constructed across all 12 potato chromosomes. Haploid-1 and -2 were derived from maternal parent PG6359. A6-26 was a highly homozygous inbred line, derived from PG6359 after five generations of selfing. The x axis indicates the physical position, and the y axis indicates the number of heterozygous SNPs (in red) and the sequencing depth (in blue).


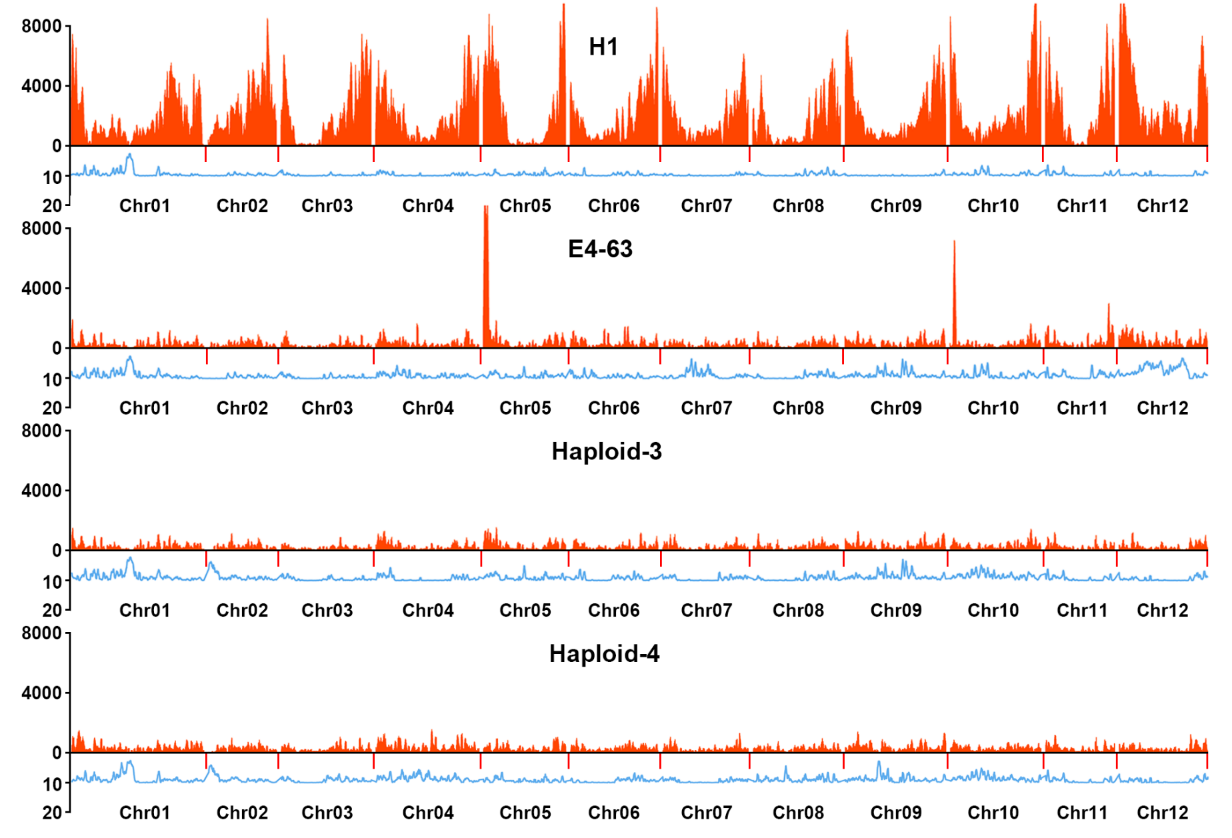


**Fig. S5. Distribution of heterozygous SNPs in haploid-3 and -4 and their corresponding maternal parent**

Chromosomal heterozygous SNP distribution maps were constructed across all 12 potato chromosomes. Haploid-3 and -4 were derived from maternal parent A6-26 × E4-63. E4-63 was a highly homozygous inbred line, derived from E86-69 after five generations of self-pollination. H1 was an F1 hybrid derived from crossing A6-26 and E4-63. The x axis indicates the physical position, and the y axis indicates the number of heterozygous SNPs (in red) and the sequencing depth (in blue).

**Table S1.** Statistical analysis of haploid induction in potato by the *stdmp* mutant

| Female | Male | Total  seeds | Seeds with weak  RFP signal | Germinated  seeds | Germination  rate | Putative  haploid | True  haploid |
| --- | --- | --- | --- | --- | --- | --- | --- |
| PG6359 | *stdmp* | 130,254 | 3,474 | 1,029 | 30% | 3 | 3 |
| A6-26 × E4-63 | *stdmp* | 81,945 | 378 | 121 | 32% | 6 | 4 |

**Table S2.** Primers used in this study

| **Primers** | **Sequence 5′-3′** | **Purpose** |
| --- | --- | --- |
| St-Qp-ACT-F | GGGATGGAGAAGTTTGGTGGTGG | RT-qPCR |
| St-Qp-ACT-R | CTTCGACCAAGGGATGGTGTAGC |  |
| St-DMP-Qp-F | TCGAGGTCCTGATGGCAAAG |  |
| St-DMP-Qp-R | TGCAATTGCCACAAACACCAA |  |
| StDMP-sp1-F | ATTGAGCAAACTAGTGAAGGAAT | Vector construction |
| StDMP-sp1-R | AAACATTCCTTCACTAGTTTGCT |  |
| St-dmp-test-F | AGAAGATTCAAAACATTTGTAAGTGCATTT | Screening for *stdmp* mutants |
| St-dmp-test-R | ACAACTGTCACGATTTATGGTGAAA |  |
| indel-F18 | CGTGGGAAGAACGTCGTG | Identification of haploid |
| indel-R18 | CATGAGTCATATGGGGTGGA |  |
